# The Relationship between Sleep Problems, Neurobiological Alterations, Core Symptoms of Autism Spectrum Disorder, and Psychiatric Comorbidities

**DOI:** 10.3390/jcm7050102

**Published:** 2018-05-03

**Authors:** Luigi Mazzone, Valentina Postorino, Martina Siracusano, Assia Riccioni, Paolo Curatolo

**Affiliations:** 1 Child Neurology and Psychiatry Unit, System Medicine Department, Tor Vergata University Hospital of Rome, Viale Oxford 81, 00133 Rome, Italy; assiariccioni@gmail.com (A.R.); curatolo@uniroma2.it (P.C.); 2 Brain and Body Integration Mental Health Clinic, 6000 East Evans Ave, Denver, CO 80222, USA; valentina.postorino86@gmail.com; 3 Child Neurology and Psychiatry Unit, Biomedicine and Prevention Department, Tor Vergata University Hospital of Rome, Viale Oxford 81, 00133 Rome, Italy; siracusanomartina@hotmail.it; 4 PHD Student in Experimental Medicine-Neuroscience, University of L’Aquila, Via Giovanni di Vincenzo, 16/B, 67100 L’Aquila, Italy

**Keywords:** sleep problems, Autism Spectrum Disorder, etiology, behavioral problems, comorbidities

## Abstract

Children with Autism Spectrum Disorder (ASD) are at an increased risk for sleep disturbances, and studies indicate that between 50 and 80% of children with ASD experience sleep problems. These problems increase parental stress and adversely affect family quality of life. Studies have also suggested that sleep disturbances may increase behavioral problems in this clinical population. Although understanding the causes of sleep disorders in ASD is a clinical priority, the causal relationship between these two conditions remains unclear. Given the complex nature of ASD, the etiology of sleep problems in this clinical population is probably multi-factorial. In this overview, we discuss in detail three possible etiological explanations of sleep problems in ASD that can all contribute to the high rate of these symptoms in ASD. Specifically, we examine how neurobiological alterations, genetic mutations, and disrupted sleep architecture can cause sleep problems in individuals with ASD. We also discuss how sleep problems may be a direct result of core symptoms of ASD. Finally, a detailed examination of the relationship between sleep problems and associated clinical features and psychiatric comorbidities in individuals with ASD is described.

## 1. Introduction

Autism Spectrum Disorder (ASD) is a neurodevelopmental condition characterized by impairments in two core domains: (1) social communication, social reciprocity; (2) restricted and repetitive patterns of behaviour, interests, or activities [1]. In addition to these core symptoms, ASD is often associated with a range of co-occurring issues, including emotional and behavioural dysregulation and psychiatric and medical comorbidities (e.g., sleep disorders). In fact, compared to typically developing (TD) children and those with other developmental disabilities, children with ASD are at an increased risk for sleep disturbances [2].

In this context, a considerable difference of sleep architecture in ASD population has been demonstrated, such as prolonged sleep latency, decreased sleep efficiency, reduced total sleep time, increased wake after sleep onset, bedtime resistance, and daytime sleepiness [3].

The International Classification of Sleep Disorders-third Edition (ICSD-3) defines more than 70 sleep disorders classified into seven major categories: insomnia disorders, sleep-related breathing disorders, central disorders of hypersomnolence, circadian rhythm sleep-wake disorders, sleep-related movement disorders, parasomnias, and other sleep disorders [4]. 

For the purpose of this overview we divided sleep disorders in dyssomnia and parasomnias. Dyssomnia may be defined as difficulty initiating or maintaining sleep that is viewed as a problem. In the ICSD-3, pediatric insomnia is included in the category of “Behavioral Insomnia of Childhood” divided in two types: sleep-onset association type and limit-setting type. Sleep-onset association type occurs when a child associates falling asleep with an action (being held or rocked), object (bottle), or setting (parents’ bed), and is unable to fall asleep if separated from that association. Limit-setting type occurs when a child stalls and refuses to go to sleep in the absence of strictly enforced bedtime limits. Parasomnias are a group of sleep disorders that involve unwanted events or experiences that occur while you are falling asleep, sleeping, or waking up. Following the ICSD-3, parasomnias are subdivided into three groups: non-rapid eye movement (NREM)-related, rapid eye movement (REM)-related, and other parasomnias.

The prevalence of sleep problems among typically developing children is around 25% [5], while in ASD population it ranges from 50 to 80% [6,7,8,9]. Malow et al. [9] investigated the prevalence of sleep difficulties in 1518 children with ASD aged 4 to 10 years using data within the Autism Speaks Autism Treatment Network Registry. These authors found that sleep problems were present in 71% of children; however, the prevalence of sleep diagnoses was less frequent (30%) in this sample. Although sleep-onset issues and insomnia seem to be the most common sleep problems reported by parents of children with ASD, night awakenings, poor sleep routines, and parasomnia are also frequent in this population [6,7,10,11,12]. 

Some studies found that age is a predictor of sleep problems: young children experience more sleep problems [13,14]. However, other reports have found that sleep problems are persistent in ASD and vary with age [15,16,17,18]. For example, Goldman et al. [16], evaluating a sample of 1859 children with ASD (aged 3–18), reported that sleep problems persist throughout the age span from early childhood through adolescence. Additionally, these authors reported that the types of problems tend to change with age. Specifically, in this study parents of younger children reported more problems with sleep anxiety, bedtime resistance, night awakenings, and parasomnias, whereas parents of adolescents reported more problems with sleep-onset, sleep duration, and daytime sleepiness.

Studies investigating clinical features (e.g., cognitive functioning, epilepsy) associated with sleep problems in children with ASD reported mixed results [19]. For example, Taylor et al. found that lower overall intellectual functioning was associated with fewer hours of sleep per night in children with ASD [20]. By contrast, other studies have found that individuals with ASD report sleep problems regardless of their cognitive level [2,21,22]. 

Moreover, it has to be noticed that sleep problems can be related and influenced by a variety of underlying medical conditions common in individuals with ASD, such as epilepsy [23]. In fact, sleep and epilepsy reciprocally affect each other: among children with epilepsy there is a higher prevalence rate of sleep disorders, in turn, seizures may be exacerbated by sleep deprivation [24]. 

**Figure 1:**
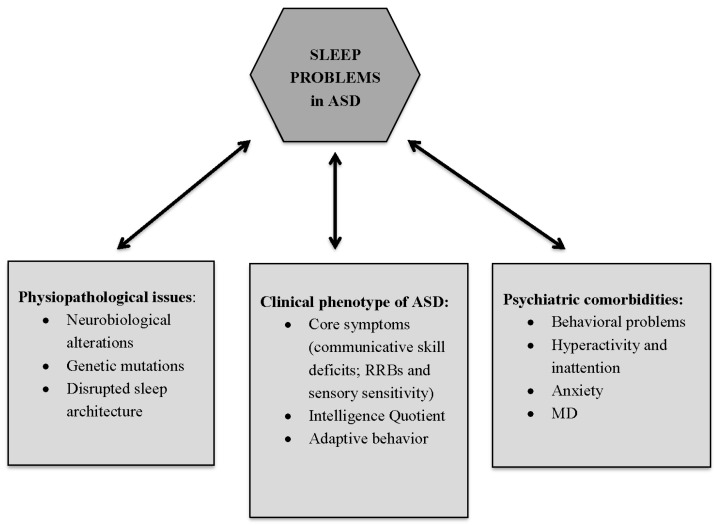
**Possible etiological explanations of sleep problems in Autism Spectrum Disorder (ASD).** (1) Sleep problems are a consequence of biological and genetic abnormalities (involving serotonin, melatonin, and GABA) and disrupted sleep architecture present in individuals with ASD; (2) Sleep problems are intrinsic to the clinical phenotype of ASD; (3) Sleep problems represents a co-occurring condition completely independent from ASD. RRBs = Restricted interests and repetitive behaviours; MD = Mood Disorders.

Although understanding the causes of sleep disorders in ASD is a clinical priority, the causal relationship between these two conditions remains unclear. The ongoing debate on the possible causes of sleep disorders in ASD is still open with three possible etiological explanations: (1) sleep problems are a consequence of biological and genetic abnormalities and disrupted sleep architecture present in individuals with ASD; (2) sleep problems are intrinsic to the clinical phenotype of ASD; (3) sleep problems represents a co-occurring condition completely independent from ASD [7] (Figure 1).

In this overview we will summarize, evaluate, and discuss the current literature related these three possible etiological explanations of sleep problems in ASD. Specifically, we will examine how neurobiological alterations, genetic mutations, and disrupted sleep architecture can cause sleep problems in individuals with ASD. We will also discuss how sleep problems may be a direct result of core symptoms of ASD. Finally, a detailed examination of the relationship between sleep problems and associated clinical features and psychiatric comorbidities in individuals with ASD will be described.

## 2. Are Sleep Problems a Consequence of Biological Abnormalities, Genetic Mutations, and Disrupted Sleep Architecture Present in Individuals with ASD?

Neurobiological alterations, genetic mutations, and disrupted sleep architecture can play a role in the onset and exacerbation of sleep problems present in individuals with ASD [7,19,25,26]. Disrupted expression of several neurotransmitters, such as serotonin, melatonin, and gamma-aminobutyric acid (GABA), that have been described in the cause of ASD are also implicated in the regulation of sleep-wake cycle. 

Serotonin or 5-hydroxytryptamine (5-HT) is a monoamine neurotransmitter involved in the body’s sleep-wake cycle [27,28,29]. Dysregulation of the serotonergic signaling system, such as increased levels of blood serotonin, altered serotonin synthesis and degradation, and genetic mutations in serotonin pathways (e.g., transporter gene SLC6A4), have been reported in population with ASD [30]. It is well known that the alteration of the 5-HT system play an important role in the regulation of the sleep-wake cycle. Impairment of the circadian rhythm can impact on the brain development. In this context, Geoffray et al. (2016) highlight a possible bidirectional relationship between circadian rhythms and ASD [28]. Specifically, the expression of specific molecules that are implied in the etiology of ASD (e.g., neurexins and neuroligins), can be impaired by circadian rhythm dysregulation during the critical period of brain plasticity. Furthermore, these authors suggest that ASD vulnerability factors and negative environmental conditions (e.g., nutrition, stress) may enhance circadian rhythms disorders [28].

Melatonin is a naturally occurring hormone involved in coordinating the body’s sleep-wake cycle. Several studies have described abnormal melatonin regulation in ASD [31,32,33,34]. For example, Tordjman et al. [33], measuring levels of a major metabolite of melatonin (i.e., 6-sulphatoxymelatonin) in 49 children and adolescents with ASD and 88 age-and-sex-matched controls, found that nocturnal production of melatonin was significantly reduced in autism. Similarly, genetic mutations in the melatonin pathways have been reported [35,36,37,38]. In more detail, the production of melatonin involves different enzymatic reactions, such as acetylserotonin O-methyltransferase (ASMT) enzyme which converts serotonin to melatonin. Melke et al. [37] have reported that a decreased expression of the ASMT transcript is correlated with decreased blood melatonin levels in individuals with ASD. These authors suggested that this deficit in melatonin might be responsible for sleep problems in ASD, and specifically circadian abnormalities. However, in a large sample, Toma et al. [35] did not find differences in ASMT variants in ASD compared to controls. 

GABA is the principal inhibitory neurotransmitter in the central nervous system. Nelson et al. [39] identified a mutation containing GABA genes in chromosome 15q in individuals with ASD. This mutation may result in a disruption of GABA inhibitory functions and in turn lead to sleep problems in ASD. 

Sleep architecture represents the cyclical pattern of sleep as it shifts between the different sleep stages, including non-rapid eye movement (NREM) and rapid eye movement (REM) sleep. It may change with age and can have an impact on the quality of life. For example, it is known that that during the physiological development, NREM sleep gradually increase while REM sleep decrease, resulting in a lighter sleep; thereby, it might become easier to awaken throughout the night and harder to fall and stay asleep at night. Disrupted sleep architecture, including increased REM density, reduction of REM sleep, or longer sleep latency, has been also described in individuals with ASD [3,21,40,41,42,43,44,45]. For example, Bruni et al. [44] evaluated sleep architecture and NREM sleep alterations by means of cyclic alternating pattern (CAP) in 18 children with ASD compared to 12 controls, and found peculiar CAP modifications in children with ASD with a correlation of the quantification of sleep electroencephalographic (EEG) oscillations with the degree of mental ability/disability. In another study, Miano et al. [46] showed alterations of NREM sleep in children with ASD. A recent prospective longitudinal study in 73 children with ASD found that sleep duration, evaluated with parental questionnaires, in children with ASD is reduced from 30 months of age and this reduction persists until adolescence [3]. However, another study showed that young adults with Asperger Syndrome have a similar polysomnographic profile compared with controls [42]. Differences in methodology and samples can explain inconsistent results. 

Future research in this area should use more rigid diagnostic criteria and definitions of sleep problems. For example, the use of both objective (i.e., polysomnography, actigraphy, and videosomnography) and subjective measures (i.e., parent-report, sleep diaries) of sleep may reduce the discrepancy in the results [47]. Furthermore, it has to be noted that subjective measures of sleep, such as clinical interviews, self-report questionnaires, and checklists, have been designed and standardized to investigate sleep disorders referring to the general population and they may not be appropriate for individuals with ASD. For example, one commonly employed sleep questionnaire for children is the Children’s Sleep Habits Questionnaire (CSHQ) [48]. This questionnaire has been used in children ranging from preschool age through school age and in previous research in ASD [8,48,49,50]. Johnson et al. [51] explored the psychometric properties of the CSHQ in a sample of 310 children with ASD. These authors found that a modified version with five subscales demonstrated adequate psychometric properties in the sample of children with ASD. Therefore, psychometric development of subjective measures of sleep specifically for use with ASD is suggested in order to improve our ability to assess sleep disorders in a reliable, valid, and time-efficient manner.

## 3. Are Sleep Problems Intrinsic to the Clinical Phenotype of ASD?

A possible hypothesis on the causes of sleep problems in ASD is that these may themselves be a core feature of autism. Restricted interests and repetitive behaviours (RRBs) represent a core diagnostic feature of ASD and include repetitive and ritualistic behaviours, cognitive inflexibility, stereotypies, and insistence on sameness. Individuals with ASD often are attached to routines and rituals, such as bed time routine. Not adhering to this routine can lead to significant distress, which in turn, can delay sleep onset or cause insomnia [7,25]. On the other hand, given that transition between activities are often difficult for children with ASD, not having an established bed time routine can lead to problem behaviours (e.g., noncompliance) which, in turn, can delay bed time [52,53]. Individuals with ASD can also perseverate in activities (e.g., time-consuming rituals) before bed time, and perseveration in activities may delay bed time as well. Accordingly, excessive and repetitive cognitive activities, including intrusive thoughts, may determine physiological hyperarousal and emotional hyper-reactivity, contributing to sleep onset delay [54,55]. 

Moreover, it is possible that sleep problems can be exacerbated by deficits in communication skills. Specifically, these deficits can interfere with the child’s ability to understand when parents ask children to go to bed or to fall asleep. A recent randomized controlled trial examined the efficacy of an interactive, web-based parenting tutorial for improving children’s engagement in daily routines as well as improving children’s social communication and parenting efficacy and stress [56]. Results of this study showed that parents in the tutorial group reported significantly higher use of evidence-based instructional strategies and higher levels of child engagement during routines with time. Furthermore, they also reported significantly lower parenting stress and higher parenting efficacy, as well as higher ratings of child social communication one month after completion compared to the control group. These authors suggest that improvements in routine-specific behaviours may support early social-communication development. 

Finally, sensory over-responsivity and hyperarousal are also common characteristics in ASD and may influence sleep disorders in this clinical population [11,57,58]. For example, children with ASD often experience greater sensitivity to environmental stimuli and difficulties in regulating arousal and these may lead to sleep-onset delay and insomnia. Reynolds et al. [57], in a sample of 27 children with ASD, found that sleep problems were correlated with sensory problems. Accordingly, a recent study examining the relationships among sleep problems, sensory over-responsivity, and anxiety in 1347 children with ASD found that children with ASD who have sensory over-responsivity seem to be particularly predisposed to sleep problems [58]. Furthermore, Veatch et al. [59] found that sleep duration was negatively correlated with the severity of core symptoms of ASD in a sample of 2714 children with ASD, using data within the Simons Simplex Collection. The results of this study also showed that short sleep duration was positively correlated with Intelligence Quotient.

These preliminary findings indicate the need for further research in this field. In fact, while there is evidence supporting this scenario, if sleep problems are part of having ASD, it is not clear why not all individuals with ASD have sleep problems. For example, longitudinal studies investigating the relationship between the severity of repetitive behaviour or sensory over-responsivity in people with ASD and sleep patterns evaluated through objective measures can help to shed light on this issue. 

## 4. ASD, Sleep Problems, and Other Psychiatric Features

A third possible scenario is that sleep problems are a condition completely independent from ASD. In this context is important to examine the relationship between sleep disorders and associated psychiatric comorbidities in individuals with ASD. In fact, it is possible that sleep problems may worsen associated psychiatric symptoms, such as disruptive behaviours or aggression [21,59,60,61,62,63,64,65]. By contrast, it can also be possible that associated psychiatric features, such as attention deficit hyperactivity disorder (ADHD), may worsen sleep disorders present in individuals with ASD [30,53,61,66,67,68,69,70]. 

### 4.1. Behavioral Problems

Children with ASD often exhibit a range of behavioral problems including disruptive behaviors, tantrums, aggression, self-injury, hyperactivity, impulsiveness, and noncompliance [71]. These behavioral problems increase parental stress and adversely affect family quality of life [72,73].

Several studies have reported that sleep problems may worsen behavioral problems in individuals with ASD [21,49,60,61,62,64,65,70,74]. For example, Fadini et al. [62], examining the relationship between sleep and behavior in a sample of 45 children with ASD, found that sleep disturbances were associated with thought and total behavioral problems using the Child Behavior Checklist (CBCL). Similarly, Sikora et al. [74], in a sample of 1193 children with ASD, found that children with ASD and sleep disturbances had more internalizing and externalizing behavior problems, and poorer adaptive skill development than children with ASD without sleep problems. Park et al. [75] examined sleep disorders and their correlates and comorbid psychopathology in 166 children with ASD compared to 111 unaffected siblings. These authors found that children with ASD and sleep disturbances were more likely to engage in aggressive behaviors, internalizing, externalizing, and total behavioral problems compared to those without sleep problems. 

These results support the idea that improving young children’s sleep has the potential to decrease children’s behavioral problems, and in turn may decrease parental stress and improve family functioning. A recent report describing a practice pathway for the identification, evaluation, and management of insomnia in children and adolescents with ASD, suggests that pharmacological therapy (i.e., melatonin) may be indicated in certain situations [76]. Behavioral interventions, such as sleep hygiene have been found to be effective in treating sleep difficulties in children with ASD [77,78,79]. Accordingly, parents of children with ASD and sleep problems can make use of tools to manage these disturbances. A parent-training intervention which emphasizes the role of parents teaching them strategies to prevent or respond to these behaviors warrants interest as a front-line intervention model. For example, in a pilot randomized controlled study Johnson et al. [80] demonstrated the feasibility and the initial efficacy of a parent training program targeting sleep disturbances in a sample of well-characterized young children with ASD. 

### 4.2. Hyperactivity and Inattention

Children with ASD often display hyperactivity and inattentive symptoms, and it has been reported that about 30% of them meet diagnostic criteria for a comorbid ADHD [81]. Several studies have found that children with ADHD experience high level of sleep problems including variable sleep patterns, sleep-onset delay, insomnia, and consequently daytime sleepiness [26]. In line with these results, it has been described that ADHD symptoms may worsen sleep disturbances. For example, high level of arousal that can be caused by hyperactivity may delay sleep onset, and over time may cause insomnia [61]. Therefore, the presence of these symptoms may increase the likelihood of experiencing sleep disorders also in individuals with ASD. Devincent et al. [82] investigating the relationship between sleep problems and psychiatric symptoms, found that children with ASD and sleep difficulties had also higher rates of ADHD compared to children without sleep problems. Similarly, Goldman et al. [60], evaluating the possible effect of parental sleep concerns on sleep architecture through an objective measures (i.e., actigraphy), found that ASD poor sleepers were reported to have more inattention, hyperactivity, and restricted/repetitive behaviors. 

Medications often used to treat ADHD symptoms, such as methylphenidate, are known to disrupt sleep and might also contribute to enhance sleep problems. For example, in a randomized, double-blind crossover study, Sangal et al. [83] found that methylphenidate resulted in increased sleep-onset latency significantly more than did atomoxetine in 85 children with ADHD. Therefore, a comprehensive diagnostic evaluation which can include psychological, physical, and medication management investigations is always recommended in order to shed light on the cause of sleep issues in children with ASD. 

### 4.3. Anxiety and Mood Disorders 

Anxiety has been often described in children and adolescents with ASD, and a recent meta-analysis reported that 39.6% of young people with ASD had clinically elevated levels of anxiety or at least one anxiety disorder [84]. Studies have reported that anxiety is associated with psychological hyperarousal, which in turn can lead to difficulty in falling asleep and insomnia [85,86]. Indeed, it is possible that anxiety leads to intrusive thoughts and worries during the pre-sleep time, thereby interfering with sleep onset. 

Similarly, mood disorders, such as depressive or bipolar disorders, have frequently been reported in individuals with ASD [81,87]. These disorders have been also reported to be associated with hyperarousal or excessive cognitive activity. For example, it is known that individuals with bipolar disorder can experience reduced need of sleep [88].

Several studies have reported that anxiety and mood disorders may worsen sleep disorders in typically developing children [58,88,89,90,91]. These studies have also found that anxiety is linked to the development of insomnia, whereas insomnia can lead to develop depressive symptoms [92,93]. 

A growing body of evidence suggests that anxiety and mood symptoms are associated with sleep problems also in individuals with ASD [14,15,42,58,70,94,95,96]. For example, Nadeau et al. [70], investigating association of sleep and behavioral issues in 102 children with ASD and comorbid anxiety, found that the number of sleep problems was associated with internalizing and externalizing symptoms, as well as anxiety symptoms. Similarly, a study on 477 children with autism showed that parent reported sleep difficulties increased with severity of parent-reported anxiety and mood symptoms [14]. 

Previous studies in children with anxiety have demonstrated that treatment of these symptoms resulted in an improvement of sleep problems [88,89]. Nadeau et al. [70] in a subset of 40 participants with ASD demonstrated a reduction of sleep disturbances following completion of a family-based cognitive–behavioral therapy (CBT). Therefore, it is possible that the reduction of these symptoms may reduce sleep problems also in individuals with ASD. However, further research examining how pharmacological or psychological treatment protocols adapted to meet the needs of youth with ASD and anxiety can reduce sleep problems are still needed. 

## 5. Conclusions

Studies have highlighted that children with ASD often experience sleep problems. However, the relationship between these conditions still remains unclear. Sleep disturbances may also contribute to stress in families of children with ASD, and may also worsen problem behaviors in this clinical population [96]. Therefore, understanding the possible etiological causes of sleep problems is a clinical priority. Clinicians should be aware that, given the complex nature of ASD, the etiology of sleep problems in ASD is probably multi-factorial. In this overview, we discussed in detail three possible etiological explanations of sleep problems in ASD that can all contribute to the high rate of these symptoms in ASD. Future research using objective measure of sleep could help to shed light on the relationship between these two conditions. Longitudinal studies that could identify patterns of sleep and could discern between the bidirectional impaction of sleep problems and associated clinical features and psychiatric comorbidities in individuals with ASD are needed. Finally, these researches could help to develop and identify the most appropriate intervention and treatment strategies.
